# Surgical treatment of gastric adenocarcinoma: what factors influence the prognosis?

**DOI:** 10.1590/0102-67202025000035e1904

**Published:** 2025-10-27

**Authors:** Carlos Roberto NAUFEL, Anelyse Pulner AGULHAM, Beatriz Alvarez MATTAR

**Affiliations:** 1 Hospital Universitário Evangélico Mackenzie, General Surgery Department – Curitiba (PR), Brazil.; 2 Faculdade Evangélica de Medicina Mackenzie Evangelical School of Medicine – Curitiba (PR), Brazil.

**Keywords:** Stomach neoplasms, Gastrectomy, Chemotherapy, Survival analysis, Neoplasias gástricas, Gastrectomia, Quimioterapia, Análise de sobrevida

## Abstract

**Background:**

Gastric cancer is the fifth most common cancer in the world and the fourth leading cause of deaths in oncology.

**Aims:**

The aim of this study was to investigate the factors that affect the survival of patients with gastric adenocarcinoma undergoing gastrectomy in a tertiary center in South Brazil.

**Methods:**

This was a cross-sectional, observational, and retrospective study of 82 patients with gastric adenocarcinoma who underwent surgical treatment from January 2018 to August 2022. Epidemiological and prognostic factors were analyzed, such as age, sex, tumor location in the stomach, lymph node invasion, tumor extension, angiolymphatic invasion, tumor differentiation, presence of distant metastasis, compromised surgical margins, adjuvant or neoadjuvant chemotherapy, and patient survival time.

**Results:**

Of the 82 patients, 41.5% died during the follow-up period, with a maximum follow-up period of 56 months. The median time to death was 22.4 months after performing the gastrectomy. Advanced age (hazard ratio [HR]=2.76; p=0.014, p<0.05), location of the tumor in the fundus of the stomach (HR=2.77; p=0.020, p>0.05), and presence of distant metastasis (HR=2.13; p=0.039) showed a significant negative impact on survival in the multivariate analysis. On the other hand, patients undergoing adjuvant (HR=5.33; p=0.001, p<0.05) or neoadjuvant (HR=3.36; p=0.006, p<0.05) chemotherapy had a positive impact.

**Conclusions:**

The present study demonstrated that survival in patients with gastric adenocarcinoma is negatively influenced by advanced age, tumor location in the fundus of the stomach, and the presence of distant metastases, in contrast to the positive impact of performing adjuvant or neoadjuvant chemotherapy.

## ARTICLE HIGHLIGHTS

Gastric cancer (GC) is the fifth most common malignancy and the fourth leading cause of cancer-related deaths globally.

Survival in patients with gastric adenocarcinoma is negatively influenced by advanced age, tumor location in the fundus of the stomach, and the presence of distant metastases.

In contrast, survival is positively influenced by the administration of adjuvant or neoadjuvant chemotherapy.

## Summary

**CENTRAL MESSAGE:**

Gastric cancer (GC) is the fifth most common malignancy and the fourth leading cause of cancer-related deaths globally. The most common histological type is adenocarcinoma, representing more than 90% of the cases. Therapeutic options are chosen according to the staging and include surgical treatment, such as partial or total gastrectomy, with D2 lymphadenectomy, which is considered the gold standard with curative intent. Neoadjuvant, adjuvant, or perioperative chemotherapy is indicated for patients with stage 1B or higher disease, in addition to molecular targeted therapies, immunotherapy, and radiotherapy.

**PERSPECTIVES:**

Advanced age, the presence of distant metastases, and the location of the tumor in the fundus of the stomach have a negative impact on the survival of patients affected by gastric adenocarcinoma, as demonstrated by both univariate and multivariate analyses. On the other hand, adjuvant and/or neoadjuvant chemotherapy has a positive impact. In this context, accurate tumor localization is essential, as tumors in different locations differ in their clinicopathological characteristics and prognosis. Furthermore, the importance of early diagnosis and ideal therapeutic planning becomes evident.

## VISUAL ABSTRACT

**Figure:**
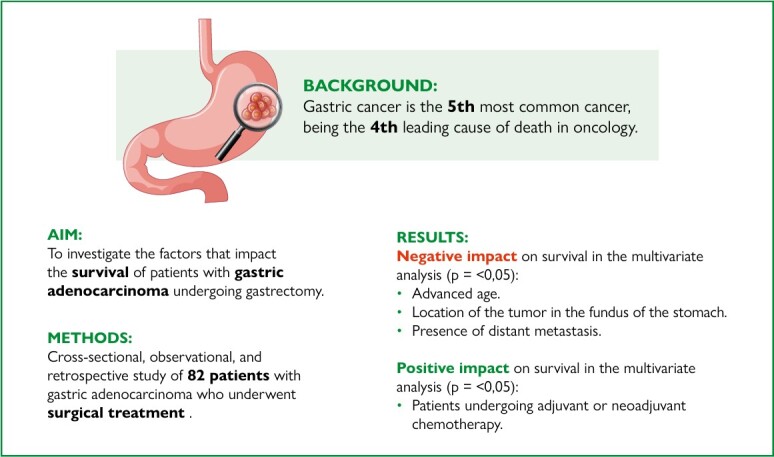

## RESUMO

**Racional:**

O câncer gástrico é o quinto câncer mais diagnosticado e a quarta principal causa de morte relacionada ao câncer em todo o mundo.

**Objetivos:**

Investigar os fatores que afetam a sobrevida de pacientes com adenocarcinoma gástrico submetidos à gastrectomia em um centro terciário no sul do Brasil.

**Métodos:**

Estudo transversal, observacional e retrospectivo, de 82 pacientes com adenocarcinoma gástrico, submetidos a tratamento cirúrgico, no período de janeiro de 2018 a agosto de 2022. Foram analisados fatores epidemiológicos e prognósticos, como idade, sexo, localização do tumor no estômago, invasão linfonodal, extensão tumoral, invasão angiolinfática, diferenciação do tumor, presença de metástase à distância, margens cirúrgicas comprometidas, quimioterapia adjuvante ou neoadjuvante e tempo de sobrevida dos pacientes.

**Resultados:**

Dos 82 pacientes, 41,5% foi à óbito no período de seguimento, sendo o período máximo de acompanhamento de 56 meses. A mediana de tempo até o óbito foi de 22,4 meses, após a realização da gastrectomia. Idade avançada (HR=2,76; p=0,014, p<0,05), localização do tumor no fundo do estômago (HR=2,77; p=0,020) e presença de metástase à distância (HR=2,13; p=0,039, p>0,05) apresentaram um impacto negativo significativo na sobrevida, na análise multivariada. Por outro lado, os pacientes submetidos à quimioterapia adjuvante (HR=5,33; p=0,001, p<0,05) ou à neoadjuvante (HR=3,36; p=0,006, p<0,05) obtiveram um impacto positivo.

**Conclusões:**

O presente estudo demonstrou que a sobrevida em pacientes com adenocarcinoma gástrico é influenciada negativamente por idade avançada, localização do tumor em fundo do estômago e presença de metástases à distância. Em contraste com o impacto positivo da realização de quimioterapia adjuvante ou neoadjuvante.

## INTRODUCTION

 Gastric cancer (GC) is the fifth most common malignancy and the fourth leading cause of cancer-related deaths globally^26^. The most common histological type is adenocarcinoma, representing more than 90% of the cases. 

 Carcinogenesis has a multifactorial etiology, involving genetic and environmental factors, such as *Helicobacter pylori* (HP) infection, smoking, previous gastric surgeries, and a high-sodium diet^9^. 

 The diagnosis of GC is often delayed because most patients are asymptomatic in the early phases of the condition. Furthermore, when present, symptoms tend to be nonspecific, including gastrointestinal tract symptoms and systemic symptoms^30^. 

 The tumor, node, and metastasis (TNM) staging system is the internationally accepted standard for GC classification. The initial evaluation includes computed tomography (CT) scans of the chest, abdomen, and pelvis and anatomopathological exams, to define location and staging^28^. 

 Therapeutic options are chosen according to the staging and include surgical treatment, such as partial or total gastrectomy with D2 lymphadenectomy, which is the gold standard with curative intent. In selected cases, palliative surgery, cytoreductive surgery, and endoscopic resection may also be used^8,25^. Neoadjuvant, adjuvant, or perioperative chemotherapy (CT) is indicated for patients with stage 1B or higher disease, in addition to molecular targeted therapies, immunotherapy, and radiotherapy^19,24^. 

 The incidence and mortality from GC have been decreasing for several decades. However, despite advances in treatment strategies, international studies show that the survival rate remains low^1,2,16^ . 

 Therefore, it is important to analyze the factors that influence the survival of patients with GC, including age, sex, tumor location, histological type, staging, if gastrectomy was performed, number of resected and affected lymph nodes, and presence of distant metastases^7,11,17 ,18^. Furthermore, the identification of risk factors, early diagnosis, and rapid referral to specialized care are essential for a better therapeutic result and a better prognosis in GC^26^. 

 The aim of this study was to investigate factors that impact the survival of patients with gastric adenocarcinoma undergoing surgical treatment. 

## METHODS

 This cross-sectional, observational, and retrospective study, based on medical records of patients who underwent surgical treatment for GC, was conducted in a tertiary hospital in Paraná, Brazil, from January 2018 to August 2022. 

 The Institutional Ethics Committee of the Hospital Universitário Evangélico Mackenzie, Mackenzie Evangelical School of Medicine, approved this study, and it was registered online (plataformabrasil.saude.gov.br; CAAE: nº 61616322.4.0000.0103). 

 The total casuistic comprised 158 patients who underwent partial or total gastrectomy, with or without lymphadenectomy. A total of 76 patients were excluded due to the absence of neoplastic disease in the surgical specimen, the presence of primary neoplasia in another site, or the performance of gastrectomy for other causes. The final case series included 82 patients. 

 The following demographic data were collected: age and sex, whether lymphadenectomy had been performed, number of lymph nodes resected and affected by the disease, location of the tumor in the stomach, presence of angiolymphatic and wall invasion (according to the histopathological studies), presence of distant metastasis, administration of adjuvant or neoadjuvant chemotherapy, and patient survival. 

 The data were analyzed using the Stata/SE v.14.1 computer program (StataCorp LP, USA). Results of quantitative variables were described by mean, standard deviation, median, minimum, and maximum. For categorical variables, absolute and percentage frequencies were presented. Survival time was described using Kaplan-Meier curves. For univariate and multivariate analyses of factors associated with survival, Cox regression models were adjusted, considering the selection of variables using the stepwise backward approach (probability of 0.05 for entry and 0.10 for removal). The significance of the variables was assessed using the Wald test, and the estimated association measure was the hazard ratio (HR) with 95% confidence intervals. Values of p<0.05 indicated statistical significance. 

## RESULTS

 The average age of the patients was 62.8 years, with 67% of the sample being male. The antrum was the region of the stomach most affected by the tumor (54.9%). 

 Histopathological studies showed that 58.5% of the patients had poorly differentiated adenocarcinoma, 34.1% had moderately differentiated adenocarcinoma, and only 7.3% had well-differentiated adenocarcinoma. One-third of the sample (32.9%) had signet ring cells. The minority presented compromised margins (23.2%), and 67.1% presented angiolymphatic invasion. 

 Concerning tumor invasion of the stomach layers, the majority (31.7%) were classified as stage T4a. As for lymphatic invasion, 19.5% of lymph nodes could not be evaluated, 35.4% did not show lymph node metastasis, and 58.5% showed invasion in some lymph nodes. Among the patients in whom it was possible to analyze regional lymph nodes, there was a higher prevalence of N0 (41.5%), followed by N1 (24.4%), N2 (12.2%), N3a (14.6 %), and N3b (7.3%). The majority of the patients did not present with distant metastasis (76.8%). 

 Regarding treatment, 36.6% of the patients underwent neoadjuvant chemotherapy and 32.9% underwent adjuvant chemotherapy, with some patients possibly receiving both. Patients’ characteristics are summarized in Table 1. 

**Table 1:** General characteristics of the patients studied.

Variable/Classification		n	Results*
Age (years) (mean±standard deviation; min–max.)		82	62.8±12.6 (29–83)
Gender (%)			
	Female	27	32.9
	Male	55	67.1
Fundus (%)			
	No	71	86.6
	Yes	11	13.4
Corpus (%)			
	No	49	59.8
	Yes	33	40.2
Antrum (%)			
	No	37	45.1
	Yes	45	54.9
Total lymph nodes resected (median; min–max)		82	22 (3–64)
Number of lymph nodes affected (median; min–max)		82	1 (0–34)
Lymph nodes could not be assessed (%)			
	No	66	80.5
	Yes	16	19.5
Absence of lymph node metastasis (%)			
	No	53	64.6
	Yes	29	35.4
Regional lymph nodes (%)			
	No	34	41.5
	Yes	48	58.5
	None	34	41.5
	N1 (1–2)	20	24.4
	N2 (3–6)	10	12.2
	N3a (7–15)	12	14.6
	N3b (>16)	6	7.3
Surgical margin compromised (%)			
	No	63	76.8
	Yes	19	23.2
Carcinoma in situ (Tis) (%)			
	No	79	96.3
	Yes	3	3.7
Invasion of the gastric wall (%)			
	None	3	3.7
	T1a	4	4.9
	T1b	8	9.8
	T2	11	13.4
	T3	16	19.5
	T4a	26	31.7
	T4b	14	17.1
Lymphovascular invasion (%)			
	No	27	32.9
	Yes	55	67.1
Distant metastasis (%)			
	No	63	76.8
	Yes	19	23.2
Differentiation (%)			
	Poorly	48	58.5
	Moderately	28	34.1
	Well	6	7.3
Signet ring cell (%)			
	No	55	67.1
	Yes	27	32.9
Neoadjuvant chemotherapy (%)			
	No	52	63.4
	Yes	30	36.6
Adjuvant chemotherapy (%)			
	No	55	67.1
	Yes	27	32.9

* Described as mean±standard deviation (min–max) or median (minmax) (quantitative variables); absolute and percentage frequency (categorical variables).

**Figure 1:**
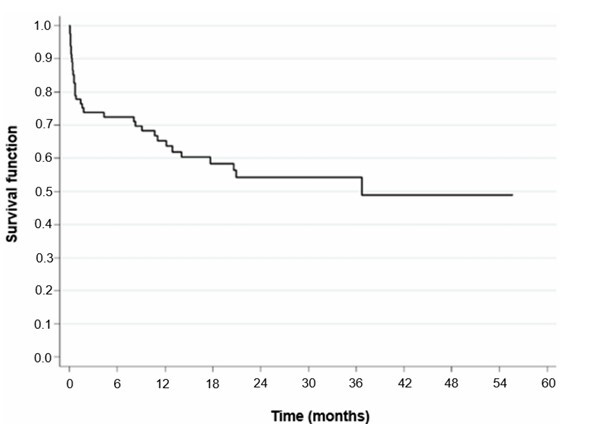
Kaplan-Meier curve for the survival of all patients in the study.

 Of the 82 patients included in the study, 48 (58.5%) died during the 56-month follow-up period. Follow-up began on the date of gastrectomy and ended either on the date of death (for patients who died) or on the date of the patient’s last visit (for patients who did not die). Figure 1 shows patients’ overall Kaplan-Meier survival curve. Table 2 presents the death percentages estimated over time (months) based on the Kaplan Meier survival analysis. 

**Table 2:** Death percentages estimated by Kaplan-Meier survival analysis over time (months).

Time (months)	% of survival
0 (gastrectomy)	100
0.5	86.5
1	77.7
3	73.9
6	72.5
12	65.3
24	54.2
36	54.2
56	48.8

**Figure 2:**
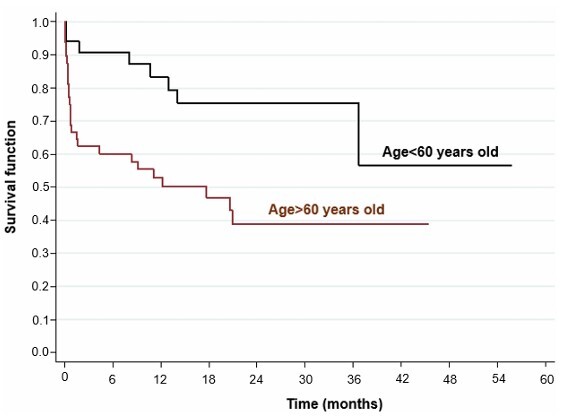
Comparison of Kaplan-Meier curves between patients aged <60 years and >60 years.

**Figure 3:**
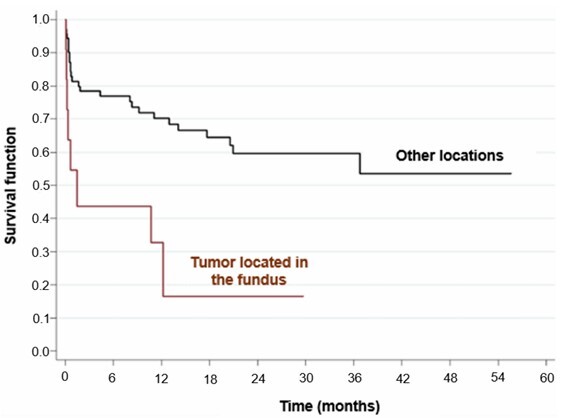
Comparison of Kaplan-Meier curves between patients with tumors located in the fundus of the stomach and tumors in other locations.

**Figure 4:**
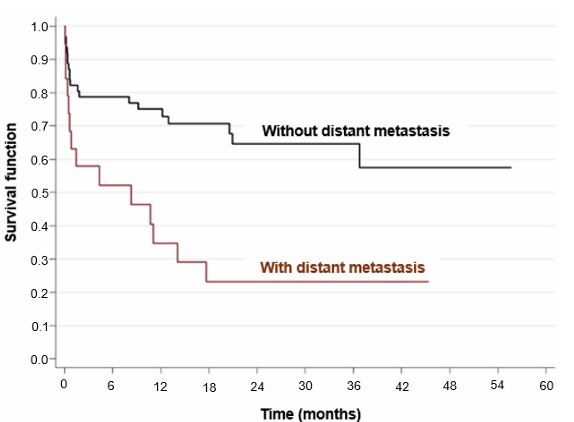
Comparison of Kaplan-Meier curves between patients with and without distant metastasis.

**Figure 5:**
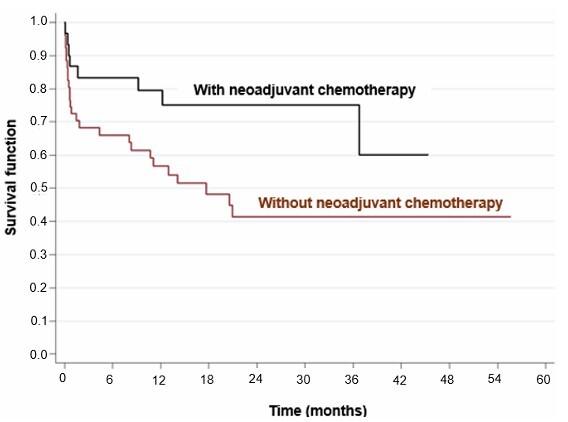
Comparison of Kaplan-Meier curves between patients who underwent neoadjuvant chemotherapy and those who did not.

**Figure 6:**
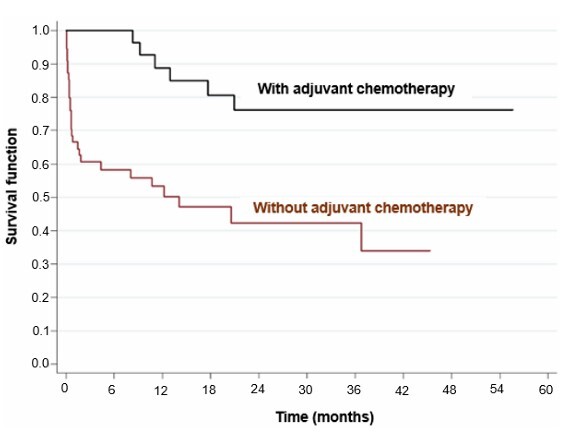
Comparison of Kaplan-Meier curves between patients who underwent adjuvant chemotherapy and those who did not.

 Univariate analysis showed that age over 60 years (HR=2.91; p=0.009, p<0.05) (Figure 2), location of the tumor in the fundus of the stomach (HR=3.49; p=0.003, p<0.05) (Figure 3), surgical margins compromised (HR=2.48; p=0.011, p>0.05), presence of distant metastasis (HR=2.97; p=0.002, p<0.05) (Figure 4), failure to perform neoadjuvant chemotherapy (HR=2.37; p=0.033, p>0.05) (Figure 5), and failure to perform adjuvant chemotherapy (HR=4.10; p=0.002, p<0.05) (Figure 6) presented an increased risk of death, as demonstrated in Table 3. 

**Table 3:** Univariate analysis by COX regression for the association of mortality with characteristics of the patients studied.

Variable/Classification		Total (100%)	Survival	Death	p-value*	HR (95%CI)
Age (years) (mean ± standard deviation; min–max)		82	58.7±13.1	68.5±9.3	<0.001	1.06 (1.03–1.10)
Age (years) (%)						
	<60	34	26 (76.5)	8 (23.5)		
	=60	48	22 (45.8)	26 (54.2)	0.009	2.91 (1.31–6.44)
Gender (%)						
	Female	27	15 (55.6)	12 (44.4)		
	Male	55	33 (60)	22 (40.0)	0.512	1.27 (0.51–2.58)
Fundus (%)						
	No	71	45 (63.4)	26 (36.6)		
	Yes	11	3 (27.3)	8 (72.7)	0.003	3.49 (1.55–7.83)
Corpus (%)						
	No	49	28 (57.1)	21 (42.9)		
	Yes	33	20 (60.6)	13 (39.4)	0.620	0.84 (0.42–1.68)
Antrum (%)						
	No	37	21 (56.8)	16 (43.2)		
	Yes	45	27 (60)	18 (40)	0.735	0.89 (0.45–1.75)
Total lymph nodes resected (median; min–max)		82	26 (4–57)	20 (3–64)	-	-
Number of lymph nodes affected (median; min–max)		82	0 (0–23)	2 (0–34)	-	-
Number of lymph nodes affected (%)†						
	=2	54	36 (66.7)	18 (33.3)		
	>2	28	12 (42.9)	16 (57.1)	0.083	1.82 (0.92–3.57)
Lymph nodes could not be assessed (%)						
	No	66	37 (56.1)	29 (43.9)		
	Yes	16	11 (68.8)	5 (31.3)	0.372	0.65 (0.25–1.68)
Absence of lymph node metastasis (%)						
	Yes	29	19 (65.5)	10 (34.5)		
	No	53	29 (54.7)	24 (45.3)	0.638	1.19 (0.57–2.51)
Regional lymph nodes (%)						
	No	34	23 (67.6)	11 (32.4)		
	Yes	48	25 (52.1)	23 (47.9)	0.300	1.46 (0.71–3.00)
	None	34	23 (67.6)	11 (32.4)		
	N1 (1–2)	20	13 (65)	7 (35)		
	N2 (3–6)	10	6 (60)	4 (40)		
	N3a (7–15)	12	5 (41.7)	7 (58.3)		
	N3b (>16)	6	1 (16.7)	5 (83.3)	-	-
Carcinoma in situ (Tis) (%)						
	No	79	46 (58.2)	33 (41.8)		
	Yes	3	2 (66.7)	1 (33.3)	-	-
Invasion of the gastric wall (%)						
	No	3	2 (66.7)	1 (33.3)		
	T1a	4	3 (75)	1 (25)		
	T1b	8	7 (87.5)	1 (12.5)		
	T2	11	10 (90.9)	1 (9.1)		
	T3	16	9 (56.3)	7 (43.8)		
	T4a	26	11 (42.3)	15 (57.7)		
	T4b	14	6 (42.9)	8 (57.1)	-	-
Surgical margin compromised (%)						
	No	63	42 (66.7)	21 (33.3)		
	Yes	19	6 (31.6)	13 (68.4)	0.011	2.48 (1.23–4.98)
Lymphovascular invasion (%)						
	No	27	20 (74.1)	7 (25.9)		
	Yes	55	28 (50.9)	27 (49.1)	0.165	1.8 (0.79–4.15)
Distant metastasis (%)						
	No (ref.)	63	43 (68.3)	20 (31.7)		-
	Yes	19	5 (26.3)	14 (73.7)	0.002	2.97 (1.50–5.91)
Differentiation (%)						
	Well	6	3 (50)	3 (50.0)		
	Moderately	28	17 (60.7)	11 (39.3)	0.507	0.65 (0.18–2.34)
	Poorly	48	28 (58.3)	20 (41.7)	0.622	0.74 (0.22–2.49)
Signet ring cells (%)						
	No	55	34 (61.8)	21 (38.2)		
	Yes	27	14 (51.9)	13 (48.1)	0.434	1.32 (0.66–2.64)
Neoadjuvant chemotherapy (%)						
	Yes	30	22 (73.3)	8 (26.7)		
	No	52	26 (50)	26 (50.0)	0.033	2.37 (1.07–5.24)
Adjuvant chemotherapy (%)						
	Yes	27	21 (77.8)	6 (22.2)		
	No	55	27 (49.1)	28 (50.9)	0.002	4.10 (1.68–10.0)

* Univariate Cox regression model, p<0.05

† Cutoff point equal to 2 obtained by fitting a ROC curve (area under the curve=0.65; p=0.021; sensitivity=56%; specificity=64%)

HR: hazard ratio; CI: confidence interval.

 The multivariate analysis shows that the variables age (HR=2.76; p=0.014, p<0.05), location in the fundus (HR=2.77; p=0.020, p<0.05), distant metastasis (HR=2.13; p=0.039, p>0.05), not performing neoadjuvant (HR=3.36; p=0.006, p<0.05) or adjuvant chemotherapy (HR=5.33; p=0.001, p<0.05) were presented as independent risk factors for mortality in patients with gastric adenocarcinoma undergoing gastrectomy (Table 4). 

**Table 4:** Multivariate analysis by COX regression to associate mortality with characteristics of the patients studied.

Variable/Classification		Total	Survival	Death	p-value*	HR (95%CI)
Age (years) (%)						
	<60	34	26 (76.5)	8 (23.5)		
	=60	48	22 (45.8)	26 (54.2)	0.014	2.76 (1.23–6.19)
Fundus (%)						
	No	71	45 (63.4)	26 (36.6)		
	Yes	11	3 (27.3)	8 (72.7)	0.020	2.77 (1.17–6.53)
Distant metastasis (%)						
	No	63	43 (68.3)	20 (31.7)		
	Yes	19	5 (26.3)	14 (73.7)	0.039	2.13 (1.04–4.36)
Neoadjuvant chemotherapy (%)						
	Yes	30	22 (73.3)	8 (26.7)		
	No	52	26 (50)	26 (50.0)	0.006	3.36 (1.40–8.04)
Adjuvant chemotherapy (%)						
	Yes	27	21 (77.8)	6 (22.2)		
	No	55	27 (49.1)	28 (50.9)	0.001	5.33 (2.02–14.1)

* Univariate Cox regression model, p<0.05.

HR: hazard ratio; CI: confidence interval.

## DISCUSSION

 This study analyzed factors that may affect the survival of patients undergoing gastrectomy for GC, such as demographic data, location of the tumor, extent of the disease, and treatment performed. 

 GC is more prevalent in males. In developed countries, GC is 2.2 times more likely to be diagnosed in men than in women^5^. In this study, men represented 67% of the sample. 

 Regarding the impact of age on the survival of patients with GC, there are several contradictory studies. In a retrospective study conducted by Liu et al., involving 317 patients with GC aged 45 years and 1344 patients aged >45 years, the authors concluded that long-term survival was significantly higher in younger patients than in elderly patients in stage I but similar between age groups in stages II and III^12^. Wang et al. performed a retrospective analysis of 3,930 patients with GC who underwent radical gastrectomy and reported that the 5-year survival rate was higher in younger patients compared to elderly patients, despite the fact that younger patients exhibited more aggressive tumors and a higher recurrence rate^29^. Another retrospective study, with 875 patients, reported that survival rates were similar between patients aged 45 years or younger (n=84) and patients older than 45 years (n=791)^20^. 

 On the other hand, a prospective study evaluated 207 patients over 6 years and concluded that survival in patients younger than 45 years tends to be lower due to the anatomopathological characteristics and the more aggressive behavior of the tumor in this age group^4,22^. In this study, the average age of patients was 62.8 years, and it was observed that the risk of death was higher in patients aged 60 years, compared to patients aged <60 years. 

 Regarding the tumor location, this study demonstrated that cancer in the fundus of the stomach is associated with a higher risk of death. This finding is similar to the conclusion of Ma et al., who reported that proximally located gastric tumors have the worst 5-year survival rate (35%), compared to 43.2% in tumors located in the middle third and 51.4% in those in the distal third^13^. In the Chinese population, Yu et al. reported a 5-year survival rate of 28% for 187 patients with proximal GC and 51% for 777 patients with distal GC^31^. Furthermore, according to Wang et al., patients with tumors located in proximal regions had a shorter survival of 91.83 months, when compared to patients with distal tumors, of 106.55 months. This indicates that distal GC is associated with better survival in the initial stage^29^. 

 GC commonly presents with non-specific symptoms, which leads to late diagnosis, often resulting in the staging of advanced or metastatic disease. Metastatic disease has a poor prognosis, and at the time of diagnosis, 35% of patients with GC have evidence of distant metastases^10,18^. 

 In the study by Riihimäki et al., which analyzed 7,559 patients with GC, 39% of the sample presented with metastasis. Patients diagnosed under 60 years of age had a median survival of 6 months, while older patients had a median survival of 3 months^21^. In clinical trials, in which more aggressive treatment is carried out, metastatic GC can result in a mean survival of 16 months, contrasting with 3–4 months in most population studies^23,27^. In this study, the presence of metastasis was a significant factor for lower survival in both univariate and multivariate analyses. 

 In the present study, the use of chemotherapy, both adjuvant and neoadjuvant, improved survival. Montagnani et al. carried out a meta-analysis and analyzed data from 33 observational studies, with a total of 1,304 patients, and concluded that the administration of adjuvant chemotherapy is related to an increase in survival^15^. 

 The CLASSIC study compared patients with stage II–IIIB tumors who underwent only D2 gastrectomy associated with adjuvant therapy (n=520) to patients who underwent only surgical treatment (n=515). The 5-year survival rate in the group receiving chemotherapy was 78%, while in the group with gastrectomy alone, it was 68%^3^. The MAGIC study compared a perioperative chemotherapy regimen –– before and after surgical treatment –– with surgical treatment alone. The perioperative regimen decreased the tumor stage and improved both progression-free survival and overall survival, with a 5-year survival rate of 36%, compared to 24% in the group undergoing gastrectomy alone^6^. 

 Miao et al. developed a systematic review and meta-analysis regarding the applicability of neoadjuvant therapy for GCs. A total of 35 studies were included, and the results of the meta-analysis indicated that neoadjuvant chemotherapy is associated with increased 5-year survival and increased diseasefree progression when compared to patients undergoing gastrectomy alone. Despite this, it is also associated with adverse pre- and postoperative effects^14^. 

 Finally, the limitations of this study, in addition to the reduced number of patients, include carrying out the study in a tertiary hospital, missing the opportunity to diagnose the disease at an early stage due to the potential lack of accessibility that may have caused a major delay in treating patients and, therefore, altering the prognosis. As it is a retrospective study, the validity of the data may also be affected by the accuracy of the information contained in the medical records. 

## CONCLUSIONS

 Advanced age, the presence of distant metastases, and the location of the tumor in the fundus of the stomach have a negative impact on the survival of patients affected by gastric adenocarcinoma, both in univariate and multivariate analyses. On the other hand, adjuvant and/or neoadjuvant chemotherapy has a positive impact. In this context, accurate tumor localization is essential, as tumors in different locations differ in their clinicopathological characteristics and prognosis. Furthermore, the importance of early diagnosis and ideal therapeutic planning becomes evident. 

## Data Availability

The datasets generated and/or analyzed during the current study are available from the corresponding author upon reasonable request.
